# The effect of maternal undernutrition on adverse obstetric outcomes among women who attend antenatal care in Gedeo zone public hospitals, cohort study design

**DOI:** 10.1186/s40795-024-00870-w

**Published:** 2024-04-22

**Authors:** Zerihun Figa, Tesfaye Temesgen, Abbas Ahmed Mahamed, Etaferahu Bekele

**Affiliations:** 1 Dilla University College of Health and Medical Science Department of Midwifery, Dilla, Ethiopia; 2 Dilla University College of Health and Medical Science Department of Emergency and Critical Care Nursing, Dilla, Ethiopia

**Keywords:** Maternal undernutrition, Preeclampsia, Obstructed labor, Prolonged labor, Preterm labor, Sepsis, Ethiopia

## Abstract

**Background:**

Undernutrition refers to an overall deficiency of nutrients due to an inadequate intake of a well-balanced diet. Undernourishment during pregnancy is an important contributor to maternal morbidity and mortality. It remains a persistent problem in developing countries, where women usually fall behind men in having access to food, health care, and education. Despite the high prevalence of maternal undernourishment, its direct impact on obstetric outcomes has not been studied in developing countries, including Ethiopia.

**Objective:**

This study aimed to assess the effect of maternal undernutrition on adverse obstetric outcomes in Gedeo zone public hospitals.

**Method:**

A cohort study design was employed in Gedeo zone public hospitals from June 30, 2022, to February 28, 2023. This study included 721 pregnant women, 237 were exposed group whereas 484 were non-exposed. A systematic random sampling technique was used to select a non-exposed group and the exposed group was selected consecutively. Both groups were followed for 7 months, from 16 weeks of gestation to 24 h of delivery. The pretested interviewer-administered questionnaire and checklist were used. EpiData 4.4.1.2.version was used for data entry and analyzed using Stata version 16 software. A modified Poisson regression model with robust standard errors was used to determine relative risk, and the statistical association was declared at a p-value ≤ 0.05. Finally, the findings were reported in figures, tables, and words.

**Result:**

The incidence of adverse obstetrics outcomes among undernourished and normally nourished mothers was hypertensive disorder during pregnancy (HDDP) (7.49% vs. 3.19%), antepartum haemorrhage (7.49% vs. 3.19%), obstructed labor (1.53% vs. 3.49%), premature rupture of the membrane (2.5% vs. 3.33%), preterm labor (6.52% vs. 6.93%), instrumental vaginal delivery (1.8% vs. 4.3%), postpartum haemorrhage (5.95% vs. 3.88%), and sepsis (3.74% vs. 1.94%). The risk of adverse obstetric outcomes among undernourished women was hypertensive disorder during pregnancy (HDDP) (aRR) = 4.07, 95%CI: 2.53–6.55), antepartum haemorrhage (APH) (aRR = 5.0, 95% CI: 2.08–12.72), preterm labor (aRR = 1.8, 95%CI: 1.23–2.62), operative delivery (aRR = 1.24, 95%C: 0.87–1.78), postpartum haemorrhage (aRR = 3.02, 95%CI: 1.91–4.79), and sepsis/chrioaminitis (aRR = 3.55, 95%CI: 1.83–6.89) times higher than normally nourished women.

**Conclusion:**

The incidence rates of hypertensive disorder during pregnancy (HDDP), antepartum haemorrhage, postpartum haemorrhage, and sepsis were higher among undernourished women than normally nourished women. Undernourished women during pregnancy have an increased risk of adverse obstetrics outcomes including hypertensive disorder during pregnancy, antepartum, preterm labor, operative delivery, postpartum haemorrhage, and sepsis/chorioamnionitis.

## Introduction

Undernutrition during pregnancy or maternal undernutrition is defined as a pregnant woman’s body mass index (BMI) of less than 18.5 kg/m2 or middle upper arm circumference (MUAC) of less than 23 cm and haemoglobin Hgb (< 11.5mmHg during the first trimester, < 10.5mmHg during the second and third trimesters) [1, 2]. It resulted from a deficiency of an important nutrient required for optimal growth and development of the fetus. During pregnancy, the physiologic changes and growing fetus increase the demand for additional nutrients. If this requirement isn’t fulfilled, the fetus will fail to gain sufficient weight and have a higher risk of mortality than healthy women [3, 4]. According to data from different studies, maternal undernutrition plays a great role in obstetric complications which may lead to life-threatening complications and morbidity [5].

Women with undernourishment also have an increased risk of obstetric complications, which can lead to maternal mortality and morbidity. The common obstetric complications associated with maternal undernourishment include preeclampsia, obstructed labor, preterm labor, premature rupture of the membrane, prolonged labor, operative delivery, and sepsis/chorioamnionitis [6]. According to data, preeclampsia results from the mediated effects of oxidative stress, inflammation, maternal endothelial dysfunction, and raised blood pressure because of maternal undernourishment [7]. Chronic undernutrition results in maternal short stature, higher cesarean delivery, cephalo-pelvic disproportion, and an increased need for assisted delivery [5].

Globally, maternal malnutrition accounts for 7% of the disease burden and contributes to at least a fifth of maternal mortality, with a higher contribution from poor pregnancy outcomes. It was widely prevalent in the regions of Southeast Asia, South America, and Africa; some countries in the region have a maternal under-nutrition prevalence as high as 35% [8]. A serious problem of maternal undernutrition is evident in most countries in sub-Saharan Africa, south-central and south-eastern Asia, and Yemen, where more than 20% of women have a body-mass index of less than 18·5 kg/m² [9].

In low- and middle-income countries, maternal malnourishment is largely the result of food insecurity, a lack of food diversity, and disease, resulting in inadequate nutrient intake and losses [10]. The linkage between maternal nutrition and child growth creates a self-perpetuating cycle of poor maternal and child growth [11]. In Sub-Saharan Africa, South-central and South-eastern Asia, more than 20% of women have a body mass index of less than 18.5 kg/m2 and this figure is as high as 40% in Bangladesh, Eritrea, and India. In countries where the prevalence of HIV infection is high, it has both a direct impact on the nutritional status of women and children who are infected and an indirect effect [12]. According to results in Ethiopia, the major maternal micronutrient deficiencies of public health importance are iodine deficiency, vitamin A deficiency, and iron deficiency [13]. And likewise, according to EDHS data disclosed in Ethiopia 18% of women are mildly anemic, 5% are moderately anemic, and 1% are severely anemic [14].

There are no findings or studies conducted to assess the effect of maternal undernutrition on adverse maternal outcomes in Ethiopia and Africa at large. Thus, this is the main reason that led us to plan and do a study on adverse maternal outcomes among undernourished women in the Gedeo zone public hospital to add knowledge in this area.

## Objectives

### General objective

To assess the effect of maternal undernutrition on adverse obstetric outcomes among ANC women in Gedeo Zone public hospitals.

### Specific objectives

To determine the incidence of adverse obstetric outcomes among ANC women in Gedeo Zone public hospitals.

To identify the effect of maternal undernutrition on adverse obstetric outcomes among ANC women in Gedeo Zone public hospitals.

## Methodology

### Study area and study period

The area of this study was the Gedeo Zone. It is one of the Zonal administrations in the southern Ethiopian region, established in 1987. It was found 365 km away from Addis Ababa, Ethiopia at an altitude of 3200 m above sea level and hilly terrain covering 1347 km². The community of the Gedeo Zone bases their lives on the agroforestry system, which has a sustainable land use system, and a large population density of over 1300 people per km² [15]. It has 8 woredas and 4 city administrations with a total population of 1,086,768 (532,516 (49%) male and 554, 225 (51%) female). In addition, there are 276 health facilities: one referral hospital, three district hospitals, 38 health centers, 146 health posts, five NGO clinics, 36 private clinics, and 47 drug vendors. Those referral hospitals and district hospitals provide full basic obstetrics services. This study was conducted from June 30, 2022, to February 28, 2023.

### **Study design**

A cohort study design was employed.

### Population

#### Source population

All pregnant mothers who came for ANC follow-up in Gedeo Zone public hospitals.

#### Study participants

The pregnant mothers who came for ANC follow-up in Gedeo Zone health facilities during the study period.

### Eligibility

#### Inclusion criteria

The pregnant mothers who came for ANC follow-up in Gedeo Zone public hospital and volunteered to participate in the study.

#### Exclusion criteria

Pregnant women who stayed for a short period in the study area (did not have enough time to complete the follow-up period).

### Sample size determination and sampling procedure

A double proportion for a finite population was used to determine sample size and calculated by Epi Info Stat, with a 95% confidence interval, a power of 80%, 1 to 2 exposed to non-exposed ratio, 1.31 a risk ratio, and 37.1% outcome of exposed from the study conducted in Bangladesh [16]. Table 1 shows the sample size n calculation using different variables required to estimate a population proportion (648 + 20% lost to follow-up = 777 study participants). Out of 777 study participants, 278 were undernourished pregnant women (the exposed group), and the remaining 499 pregnant women were normally nourished (the non-exposed group).

**Table 1:** Sample size calculation table with Epi Info Stat Calculated by factor analysis, Gedeo Zone, southeast Ethiopia, 2022/23

Variable	Outcome in exposed	Outcome in not exposed	aRR	Power	CI	Sample size	20% lost follow up	Reference
**Birth asphyxia**	29.3	10.9	2.59	80	95	180	216	[17]
**Hospital admission**	28.8	5.4	5.34	80	95	101	121	[18]
**Stunting**	36.8	31.7	1.31	80	95	648	777	[16]

**Fig. 1:**
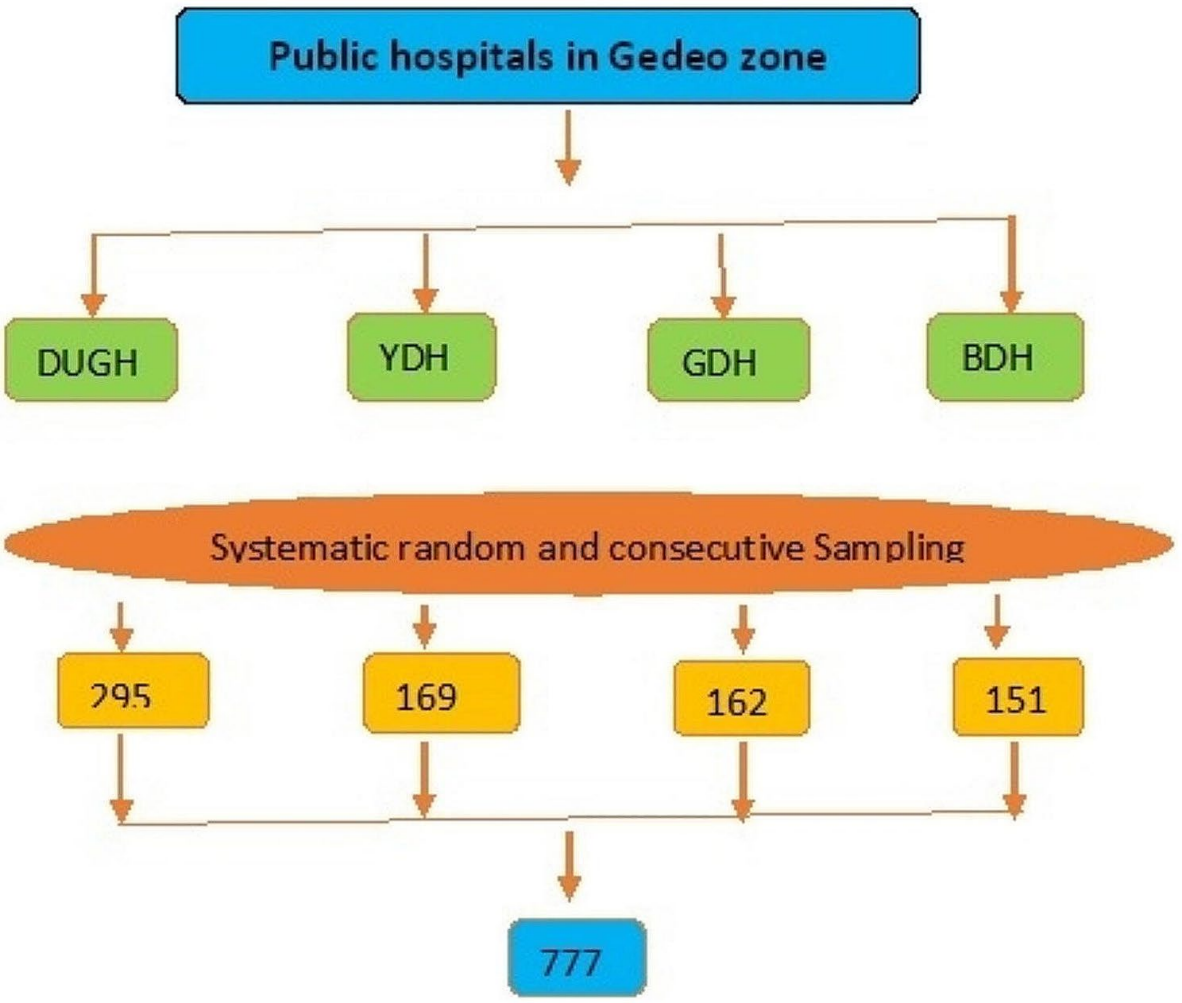
Proportionate allocation of study participants in four public hospitals of Gedeo zone public hospitals 2022/23. *Note:*DUGH: Dilla University general hospital: YDH; Yirgachefe district Hospital; GDH; Gedeb district hospital: BDH; Bule district hospital

Figure 1 shows the proportionate allocation of study participants in four public hospitals in the Gedeo zone. The study units for the exposed and non-exposed groups were selected by systematic random sampling and consecutive sampling techniques, respectively.

### Research variable

#### Dependent variables

Adverse obstetric outcomes (hypertensive disorder during pregnancy, obstructed labor, prolonged labor, preterm labor, premature rupture of the membrane, sepsis/chorioamnionitis).

#### Independent variable

Maternal under-nutrition, Socio-demographic characteristics like age, residence, marital status, religion, ethnicity, educational status, occupation, monthly income, family size, and history of medical conditions (HTN, DM, cardiac problems), Obstetric factors like the previous history of adverse obstetric and birth outcomes, the intention of pregnancy, ANC follow-up, parity, and gravidity.

### Data collection instruments and procedure

Maternal undernourishment was the primary exposure variable, which was measured by a tool developed by the principal investigator and co-investigators [4, 19, 20] and translated into Amharic and Gede’uffa versions. It had four important parts: maternal anthropometric measurement (which was measured by trained personnel, and all equipment was calibrated with standard weights and length rods), clinical measurements (from laboratory results of haemoglobin level), socio-demographic variables, past obstetric history, and obstetric outcomes.

Anthropometric and clinical measurements assessed the nutritional status of the women. During the ANC follow-up, an interviewer-administered questionnaire collected the socio-demographic characteristics and obstetric history. The obstetric outcomes information was retrieved from the medical records of the women. Two MSc midwifery supervisors and four BSc midwives participated in the data collection. The collection of the data was done in two phases: (1) during ANC follow-up, and (2) during and following delivery.

### Data quality control

Two days of training was given to the data collector and supervisor on methodology, the aim of the research, and data collection procedures. The questionnaire was prepared after a rigorous review of the literature in the English language and then translated into the local language (Gede’uffa) by the bilingual translator. The pre-test was piloted on 10% (78) outside the study area (Yirgalem General Hospital). The data was cleaned and supervised daily.

### Data analysis

EpiData version 4.4.2.1 and Stata version 16 software were used for data entry and analysis, respectively. The normality distribution test was done using the Kolmogorov-Smirnov test, and we considered a normal distribution if the p-value was ≥ 0.05. An independent t-test was used to assess the mean difference between the groups. Literature tells about the importance of using binomial and modified Poisson regression models to determine the relative risk in a cohort study with binary outcome variables. However, binomial regression has the problem of convergence, which inflates the standard error and overestimates the relative risk, while modified Poisson regression is more conservative in estimating the standard error and relative risk [21]. So that to determine the risk of developing adverse obstetrics outcomes including hypertensive disorder during pregnancy, antepartum, preterm labor, operative delivery, postpartum haemorrhage, and sepsis from undernourishment during pregnancy. A covariate significant at *p* = 0.25 in the bivariate analysis was considered under multivariate analysis. The crude and adjusted risk ratio (aRR), along with a 95% confidence interval, was estimated as the strength of the association, and a p-value ≤ 0.05 was used to declare the level of statistical significance. Finally, the data was presented in text, tables, and graphs.

### Operational definition

**Undernourished pregnant mothers:** are pregnant mothers whose upper middle arm circumference (MUAC) is less than 23 cm [1, 22, 23].

**Obstetric outcome:** was defined as the status of the mother during and after giving birth, which is identified by the most senior health professional at the respective place of delivery [18].

**Adverse obstetric outcome:** was defined as the woman delivering with any one of the obstetric-related complications, including hypertensive disorder during pregnancy (gestational hypertension, preeclampsia/eclampsia), antepartum hemorrhage (APH), obstructed labor, uterine rupture, preterm rupture of the membrane (PROM), preterm labor, instrumental delivery, operative deliveries, postpartum haemorrhage, postpartum [18].

**The exposed group:** was defined as pregnant women whose nutritional status was under-nourished during ANC follow-up.

**The non-exposed group:** was defined as pregnant women who came for ANC follow-up and had normal nutritional status.

## Result

Table 2 presents the socio-demographic characteristics of the study participants. This prospective follow-up study involved a total of 721 antenatal care women. Of this, 237 (32.87%) pregnant mothers were undernourished and the remaining 484 (67.13%) were normally nourished. The number of lost follow-ups was 56 (7.207%), with the reasons including residence change, lack of interest, and home delivery. The mean and standard deviation of maternal age with undernourishment and normally nourishment were 25.16 (5.79) and 26.307 (5.27) respectively. The mean family monthly income was higher among normally nourished women than undernourished women (2958.3 vs. 2298.01 birr). There is no difference in mean gestational age at delivery between undernourished and normally nourished women (37.20 vs. 37.72 weeks).

**Table 2:** Socio-demographic characteristics of the pregnant women attending antenatal care service in Gedeo Zone public hospital, Southern Ethiopia 2022/23

Variable/category	Maternal nutritional status		*p*-value
	Under nourished *n* = 237, *n* (%)	Normally nourished *n* = 484, n (%)	
Maternal age in years Mean (SD)	25.16878 (5.798615)	26.30785 (5.279581)	0.028
Residence			0.001
Rural	101(42.62)	144 (29.75)	
Urban	136(57.38)	340 (70.25)	
Marital status			0.098
Married	230 (97.05)	463 (95.66)	
Single	2 (0.84)	13 (2.69)	
Widowed	2 (0.84)	7 (1.45)	
Divorced	3 (1.27)	1 (0.21)	
Ethnicity			0.182
Gedeo	157 (66.24)	283 (58.47)	
Amahara	31 (13.08)	72 (14.88)	
Oromo	18 (7.59)	53 (10.95)	
Gurage	16 (6.75)	50 (10.33)	
Other	15 (6.33)	26 (5.37)	
Religion			0.126
Protestant	171 (72.15)	308 (63.64)	
Orthodox	45 (18.99)	124 (25.62)	
Muslim	14 (5.91)	38 (7.85)	
Catholic	3 (1.27)	10 (2.07)	
Other	4 (1.69)	8 (0.83)	
Mother’s educational status			0.002
No formal education	40 (16.88)	48 (9.94)	
Primary	145 (61.18)	278 (57.56)	
Secondary and above	52 (21.94)	157 (32.51)	
Partner’s educational status			0.000
No formal education	15 (6.58)	16 (3.44)	
Primary	124 (54.39)	194 (41.72)	
Secondary and above	89 (39.04)	255 (54.84)	
Mothers occupation			0.021
House wife	160 (67.51)	270 (55.79)	
Merchant	27 (11.39)	70 (14.46)	
Government employee	42 (17.72)	127 (25.24)	
Private employee	8 (3.38)	17 (3.51)	
Private employee	14 (6.11)	28 (6.02)	
Family monthly income	2298.018(1890.09)	2958.37 (2156.17)	0.000
History of chronic medical condition			0.323
Yes	10 (4.22)	29 (5.99)	
No	227 (95.78)	455 (94.01)	

**Fig. 2:**
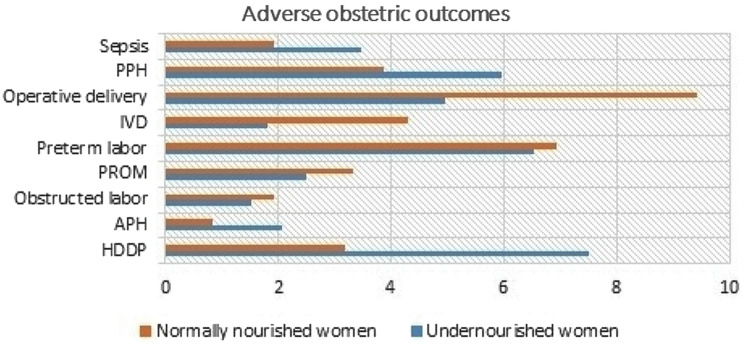
Adverse obstetric outcomes among women with undernourishment (*n* = 237) and normally nourished women (*n* = 484) in Gedeo Zone Public hospitals 2022/23. *Note:*PPH; Postpartum haemorrhage: IVD; Instrumental vaginal delivery: PROM; Premature rupture of membrane: APH; Antepartum haemorrhage: HDDP; hypertensive disorder during pregnancy

Figure 2 depicts the incidence of adverse obstetric outcomes including the incidence of hypertensive disorder during pregnancy (HDDP) (7.49% vs. 3.19%), antepartum haemorrhage (2.08% vs. 2.91%), obstructed labor (1.53% vs. 3.49%), premature rupture of the membrane (2.5% vs. 3.33%) preterm labor (6.52% vs. 6.93%), instrumental vaginal delivery (1.8% vs. 4.3%, operative delivery (5.86% vs. 9.43%), postpartum haemorrhage (5.95% vs. 3.88%), and sepsis (3.74% vs. 1.94%) among undernourished and normally nourished pregnant women.

Table 3 shows the risk of adverse obstetric outcomes associated with maternal undernutrition. To determine the effect of maternal undernutrition on adverse obstetric outcomes, a Poisson regression model with a robust standard error was applied. Thus, of the nine adverse birth outcome variables computed, half indicated the presence of a significant association with maternal undernutrition. The risk of hypertensive disorder during pregnancy (adjusted relative risk (aRR) = 4.07, 95%CI: 2.53–6.55)), antepartum haemorrhage (aRR = 5.0, 95%CI:2.08–12.72), preterm labor (aRR = 1.8, 95%CI: 1.23–2.62), operative delivery (aRR = 1.24, 95%CI:0.87–1.78), postpartum haemorrhage (aRR = 3.02, 95%CI: 1.91–4.79), and sepsis/chrioaminitis (aRR = 3.55, 95%CI:1.83–6.89) were higher among women with undernourishment during pregnancy. Whereas there is no observed significant increase in the risk of obstructed labor (aRR = 0.94, 95%CI: 0.41–2.17)), PROM (aRR = 1.56, 95%CI: 0.86–2.84), and instrumental vaginal delivery (aRR = 0.74, 95%CI: 0.37–1.46) because of maternal under-nourishment.

**Table 3:** Bivariate and multivariate analysis of modified Poisson regression of the association between maternal undernourishment and adverse obstetric outcomes in Gedeo Zone public hospitals Southern Ethiopia 2022/23

Averse obstetrics outcomes		Normally nourished women	Undernourished women	Unadjusted RR(95%CI)	Adjusted RR (95%CI)
HDDP	Yes	54 (22.78%)	23 (4.75)	4.79(3.01–7.61)	4.07(2.53–6.55) ***
	No	183 (77.22%)	461 (95.2)	1	1
APH	Yes	15 (6.33)	6 (1.24)	5.1(2.0–13.0)	5.0(2.08–12.72) **
	No	222 (93.67)	478 (98.76)	1	1
Obstructed labor	Yes	11 (4.64)	14 (3.0)	1.6(0.73–3.48)	0.94(0.41–2.17)
	No	226 (95.3)	470 (97.1)	1	1
PROM	Yes	18 (7.59)	24 (5.0)	1.53(0.84–2.76)	1.56(0.86–2.84)
	No	129 (92.41)	460 (95.0)	1	1
Preterm labor	Yes	47 (19.83)	50 (10.33)	1.91(1.33–2.77)	1.8(1.23–2.62) *
	No	190 (80.17)	432 (89.67)	1	1
Instrumental delivery	Yes	13 (5.49)	31 (6.40)	0.85(0.45–1.60)	0.74(0.37–1.46)
	No	224 (94.51)	453 (93.60)	1	**1**
Operative delivery	Yes	43 (18.14)	68 (14.0)	1.29(0.91–1.83)	1.24(0.87–1.78)
	No	194 (81.86)	416 (86.0)	1	1
PPH	Yes	43 (18.14)	28 (5.86)	3.13(1.99- 4.91)	3.02(1.91–4.79) **
	No	184 (81.86)	456 (94.21)	1	1
Sepsis	Yes	27 (11.39)	14 (2.89)	3.93(2.10–7.37)	3.55(1.83–6.89)**
	No	210 (88.61)	470 (97.11)	1	1

HDDP; hypertensive disorder during pregnancy: 1 = reference group: *=statistical significance: PPH; postpartum haemorrhage: PROM; Premature rupture of membrane: RR; relative risk: we adjusted the adverse obstetrics outcomes for age, residence, maternal educational status, partners educational status, maternal occupation, partners occupation, family monthly income, history of adverse birth outcome and chronic medical conditions

## Discussion

Optimal maternal nutrition is an important contributor to the survival of both the mother and child and promotes women’s overall health, productivity, and well-being. Currently, maternal mortality and morbidity are declining because of increased emphasis from different stakeholders on health promotion and complication prevention interventions. However, the problem remains higher in Sub-Saharan African countries and other developing countries. According to data from different studies, maternal undernutrition plays a great role in obstetric complications, which may lead to life-threatening complications and morbidity [5]. Thus, this prospective cohort study was intended to determine the incidence and risk of adverse obstetric outcomes among undernourished women.

Our study showed that the incidence of hypertensive disorder during pregnancy (HDDP) among undernourished women was 10.7% (95%CI: 8.5–13.2). This study is similar to a study conducted in the United Kingdom, 10.3% [24]. Furthermore, the risk of HDDP was 4.07 times (aRR = 4.07, 95% CI: 2.53–6.55) higher among women with undernourishment than normally nourished women. This finding is supported by a systematic review having a significant association between maternal nutritional status and HDDP (*p* < 0.001) [5]. The reason for this risk is an increase in oxidative reactions, maternal endothelial dysfunction, and raised blood pressure associated with a deficiency of different nutrients required for optimal physiologic change in the mother during pregnancy.

The incidence of premature rupture of membrane (PROM) among women with undernourishment was 2.50%, whereas the incidence rate was 3.33% among women without nourishment. The risk of premature rupture of the membrane was not significantly increased among women with undernourishment (aRR = 1.56, 95% CI: 0.86–2.84). However, some findings show an increased risk of premature rupture of the membrane among women with undernourishment in two studies from Canada [6] and 2.00 (1.09–3.67) [25]. The proposed effect of the nutritional deficiency on the premature rupture of the membrane was the altered collagen structure and interlinking architecture.

Concerning preterm labor its incidence among women with undernourishment was 6.52%. This is a similar incidence rate among women without undernourishment of 6.93%. The risk of preterm labor among women with undernourishment was 1.8 times (95% CI: 1.23–2.62) higher than women without undernourishment. This was supported by systematic review and meta-analysis, which shows 1.65 times higher risk of preterm birth among women with undernourishment [26]. The reason for the increased risk of preterm labor among women with undernourishment was associated with premature rupture of the membrane, which resulted from altered collagen structure and other interlinking structures.

The incidence of postpartum haemorrhage (PPH) among women with undernourishment was 5.96%. During the search for articles reporting the incidence of postpartum haemorrhage among women with undernourishment, we couldn’t find them. The risk of postpartum haemorrhage among women with undernourishment was 3.02 times (95% CI: 1.91–4.79) increased compared to normally nourished women. According to a study conducted in Ghana, there is a significant association between maternal undernutrition and postpartum haemorrhage (*p* < 0.001) [27]. There is a limitation of the data to compare the relative risk and underlying mechanisms of the postpartum haemorrhage.

Similarly, the incidence of sepsis or chorioaminitis among women with undernourishment was 3.74%. The risk of developing sepsis or chorioamnionitis was three times (95% CI: 1.83–6.89) increased for women with undernourishment compared to normally nourished women. The reason for the increased risk of sepsis among women with undernourishment was associated with weakness of immunity because of inadequate nutrition.

### The strength of the study

The strength of this study was that it took an adequate sample size. The prospective study design by itself is the strength. The use of modified Poisson regression is important in estimating the standard of error and relative risk because it doesn’t have the problem of convergence in estimating, so our use of this regression type increases acceptance and clarification of this finding.

### Limitation of the study

This study was done in public health institutions and didn’t include women utilizing antenatal care at the health center level or women who don’t use antenatal care services. These may affect the representativeness of the study for the entire community in the Gedeo zone.

## Conclusion

The incidence rates of hypertensive disorder during pregnancy (HDDP), antepartum haemorrhage, postpartum haemorrhage, and sepsis were higher among undernourished women than normally nourished women. Undernourished women during pregnancy have an increased risk of adverse obstetric outcomes, including hypertensive disorder during pregnancy, antepartum, preterm labor, operative delivery, postpartum haemorrhage, and sepsis/chorioamnionitis. Thus, pre-conception care should be focused on nutritional assessment and management of the undernourishment. In addition, during the intrapartum period, emphasis should be given to undernourished women.

## Data Availability

The materials and data related to this manuscript have been presented. The dataset supporting the conclusions of this article is available from the authors on reasonable request.

## Abbreviations/Acronyms

BDH: Bule district hospital
CI: Confidence Interval
DUGR: Dilla General Hospital
GDH: Gedeb District Hospital
HDDP: Hypertensive disorder during pregnancy
RR: Relative risk
PROM: Premature rupture of the membrane
OL: Obstructed labor
PL: Preterm labor
YDH: Yirga Cheffe district hospital
